# Supplementary material for: Movement patterns of the functional reach test do not reflect physical function in healthy young and older participants
Source: PLoS One. 2022 Mar 31;17(3):e0266195. doi: 10.1371/journal.pone.0266195 (PMC8970498; doi:10.1371/journal.pone.0266195)
Supplement: Attachment — Submitted filename: Response_to_Reviewers.docx [file pone.0266195.s003.docx]

**Authors’ responses to the reviewers’ comments**

**Reviewer #1:**

The authors would like to thank the reviewer for his/her constructive critique to improve the manuscript. We have made every effort to address the issues raised and to respond to all comments. The revisions are indicated in red font in the revised manuscript. Please, find next a detailed, point-by-point response to the reviewer's comments. We hope that our revisions would meet the reviewer’s expectations.

 Line 26. It is necessary that the abstract be structured so that each of its parts can be understood.

→We would like to thank the reviewer for the suggestion. Please note that we have followed the journal guidelines and formatted the manuscript according to the suggested template. Especially, the template indicates that the Abstract should be presented in an unstructured form. Moreover, please note that we have made revisions in the Abstract to improve the readability.

Line 28-30. The justification for why it is done in two age groups is not clear.

→Based on previous studies, the lack of consensus could be attributed to the different population attributes, measurement methods, and movement strategies across studies. Therefore, the participants in this study were young and older individuals so as to make a comparison of the findings. We have provided this information in the revised manuscript as follows:

“The relationship of the Functional Reach Test (FRT) value with the Center of Pressure Excursion (COPE) and physical function remains unclear, and would be influenced by different population characteristics and movement patterns used in the FRT.” (Lines 26–28)

Line 50-137. The introduction is really confusing. Too much data is provided, even a lot that could go into methods or even in discussion. Besides that, it is excessively long, causing the reader to get lost in their reading. It is recommended to redo and simplify it.

→We would like to thank the reviewer for the suggestion. Please note that we have made the appropriate revisions based on the reviewer’s suggestion.

Line 138. The justification and the objectives of the work is unclear.

→To address the issue raised by the reviewer, we have added the following part to the revised manuscript:

“Therefore, based on the aforementioned, our aim was to classify the joint movement strategies used during the FRT, using a three-dimensional motion analysis system, and to explore the relationship between the FRT value, the COPE, and physical function according to the classified pattern.” (Lines 145–148)

Line 151. I have a question. You have put in the abstract that you analyze the data in two different clusters. However, I do not see that that is contemplated or justified in this section.

→We would like to thank the reviewer for the comment. We have modified this part in the Abstract to avoid confusion. The revised part is as follows:

“The results showed that the hip strategies could be divided into two groups according to the degree of use (Small Hip Strategy, SHS Group; Large Hip Strategy, LHS Group).” (Lines 37–39)

Please note that the participants were divided into two clusters as a result of cluster analysis. We have provided this information in the “Statistical analysis” subsection as follows:

“We conducted cluster analysis based on the motion analysis data obtained during the FRT. Moreover, we performed a comparison between the clusters to investigate the influence of different movement patterns, referring to the previous study by Leroy et al [15].” (Lines 270–272)

Line 154. Perhaps providing the data for the calculation of the number of subjects required is a better fit in the statistical analysis section.

→We would like to thank the reviewer for the suggestion. Please note that we have moved the part describing the sample size calculation to the “Statistical analysis” subsection, as per the reviewer’s suggestion (Lines 259–265).

Line 160. What do you mean by 38 sides?

→We would like to thank the reviewer for the question. To avoid misunderstanding, we have revised this part as follows:

“As we aimed to compare two groups according to their movement patterns, it was predicted that more than 38 participants would be needed.” (Lines 264–265)

Line 163-5. There must be consistency in the number of decimals of the numbers provided. It is not possible that in some data there are two decimals and in others one decimal.

→The number of decimal places has been changed to two, as per the reviewer’s suggestion.

Line 167-8. This phrase is not understood. Why are 42 sides analyzed in young people and 40 in older people? Wouldn't this phrase be better in the procedure section?

→We would like to thank the reviewer for the questions. To avoid misunderstanding, we have revised this part as follows:

“Measurements were made on the left and right sides of the body; thus, the measurements were conducted on 42 and 40 sides in 21 young and 20 older participants, respectively.” (Lines 161–163)

Line 177-178. How were these markers placed?

→We would like to thank the reviewer for the question. Please note that we have provided this information as follows:

“Markers were applied to the whole body with reference to Vicon and previous studies [33].” (Lines 173–174)

Line 181-3. It is not clear where it has been described.

→We would like to thank the reviewer for the comment. To avoid misunderstanding, we have revised this part as follows:

“To calculate the detailed kinetics in the foot, a measurement method was chosen, in which one foot crossed two force plates, as Satoh et al.’s previous study described.” (Lines 178–180)

Line 188. I don't understand why some of these variables are used. They may be related, but in theory the aim of the study is to correlate FRT with COPE. Please, justify the use of the different variables relating it to what you are trying to demonstrate in the article.

→We would like to thank the reviewer for the comment. Although the measures in this study have been shown to be related to falls, each assesses a different function.

Line 242. The sentence is not clear.

→We would like to thank the reviewer for the comment. To avoid misunderstanding, we have revised this part as follows:

“The missing parts of the marker trajectory data were complemented using the gap-filling function in Vicon.” (Lines 240–241)

Line 295-6. You should indicate in the statistical analysis of the methods section whether or not the outliers are to be removed.

→As per the reviewer’s insightful suggestion, we have added this information to the revised manuscript as follows:

“We conducted cluster analysis based on the motion analysis data obtained during the FRT. Moreover, we performed a comparison between the clusters to investigate the influence of different movement patterns, referring to the previous study by Leroy et al [15]. An ascendant hierarchical clustering analysis (using Ward’s method, based on Euclidean distances [38] was used to group participants together according to the FRT value, segment displacement, and joint angle during the FRT in each group. Outliers exceeding two standard deviations were replaced by mean values. The participants in whom more than one-third of the data were outliers were excluded from the analysis.” (Lines 270–277)

Line 297. It is necessary to provide information on height, weight, etc. of the participants.

→As per the reviewer’s insightful suggestion, we have added this information to the revised manuscript as follows:

“In total, 21 young participants (42 sides; age, 25.61 ± 2.85 years; height, 1.65 ± 0.09 m; body weight, 57.10 ± 8.90 kg) and 20 older participants (40 sides; age, 73.72 ± 5.88 years; height, 1.58 ± 0.09 m; body weight, 59.64 ± 8.77 kg), respectively, were included in the analysis.” (Lines 303–307)

Line 300. It is not clear why he divided the samples into two clusters. What is it based on?
Line 303. For the same reason, it has not been explained in methods whether the results should be compared between clusters or not.

→We would like to thank the reviewer for the suggestion. Please note that we have added this information to the revised manuscript as follows:

“We conducted cluster analysis based on the motion analysis data obtained during the FRT. Moreover, we performed a comparison between the clusters to investigate the influence of different movement patterns, referring to the previous study by Leroy et al [15]. An ascendant hierarchical clustering analysis (using Ward’s method, based on Euclidean distances [38] was used to group participants together according to the FRT value, segment displacement, and joint angle during the FRT in each group. Outliers exceeding two standard deviations were replaced by mean values. The participants in whom more than one-third of the data were outliers were excluded from the analysis.” (Lines 270–277)

Line 375. The discussion is not clear. The first paragraph does not clearly answer the objectives of the study (they need to be clarified in the introduction). In turn, many of the results already presented in this section are repeated in this section. It would be advisable to redo and synthesize this section.

→We would like to thank the reviewer for the suggestion. Please note that we have added sentence to improve readability:

“In this study, we analyzed the FRT movement and measured physical function in both young and older participants to investigate the influence of different movement patterns.” (Lines 383–384)

Similar to previous works, we have included a summary of the results at the beginning of the Discussion section. For this reason, we did not consider it to be repetitive.

Line 381. It remains unclear why to split into two clusters.

→We would like to thank the reviewer for the suggestion. We have added the following parts to avoid confusion:

“We conducted cluster analysis based on the motion analysis data obtained during the FRT. Moreover, we performed a comparison between the clusters to investigate the influence of different movement patterns, referring to the previous study by Leroy et al [15]. (Lines 270–273)

“In this study, we analyzed the FRT movement and measured physical function in both young and older participants to investigate the influence of different movement patterns.” (Lines 383–384)

“Accordingly, based on the results of a previous [15] and the present study, we proceeded to analyze the results obtained from two groups, in which a different hip strategy was used in the FRT (Cluster 1, Small hip strategy [SHS]; Cluster 2, LHS). Then, we showed that the FRT values of the older SHS group were correlated with the COPE and physical function assessments, such as the TGS and the FTSST.” (Lines 389–394)

Line 394. It is recommended not to use the term cluster so much in the discussion, perhaps using what each implies would make it easier to understand this section. You should make it clearer in the methods section what each cluster implies.

→We would like to thank the reviewer for the suggestion. The clusters were distinguished based on the results of comparisons between the clusters. This information is provided in the Discussion section as follows:

“Accordingly, based on the results of a previous [15] and the present study, we proceeded to analyze the results obtained from two groups, in which a different hip strategy was used in the FRT (Cluster 1, Small hip strategy [SHS]; Cluster 2, LHS). Then, we showed that the FRT values of the older SHS group were correlated with the COPE and physical function assessments, such as the TGS and the FTSST.” (Lines 389–394)

Line 434. Although I understand this part of the discussion, having not explained it well in methods, then I do not see sense in this part of the discussion. If different variables are to be considered with respect to the FRT, I would even consider modifying the title to indicate exactly what is being investigated.

→As per the reviewer’s insightful suggestion, we have revised the title as follows:

“Movement patterns of the functional reach test do not reflect physical function in healthy young and older participants”

487. Perhaps this would go better in limitations.

→As we were referring to a limitation of the FRT, not to a limitation of this study, this information was added to the Discussion section.

Figure 2 and 3. Improving the image quality is recommended.

→We have improved the quality of these images, as per the reviewer’s suggestion.

**Reviewer #2:** This is very well written, and clearly laid out. I could reproduce your study. Your findings are valuable for rehabilitation clinicians and researchers. My biggest criticism is with the title. it could already indicate the result and highlight this as a must-read for rehabilitation clinicians.

→The authors would like to thank the reviewer for his/her constructive critique to improve the manuscript. We have made every effort to address the issues raised and to respond to all comments. The revisions are indicated in red font in the revised manuscript. Please, find next a detailed, point-by-point response to the reviewer's comments. We hope that our revisions would meet the reviewer’s expectations.

As per the reviewer’s insightful suggestion, we have revised the title as follows:

“Movement patterns of the functional reach test do not reflect physical function in healthy young and older participants”

Your abstract and introduction are clear. The abstract has no misleading statements and lays out the study well. The introduction lays out the argument in a logical manner. Lines 65-73 in particular drew me into your subject matter. There was a good scope of literature. It is true that this test and falls prevention have been around for a long time, so there was a wide range of dates, but recent work balanced the older material.

・Methodology - please include a 12 months retrospective falls diary for your older participants. Even if it is zero falls, this is important data. It may affect your results if there are falls.

→All the elderly in this study could walk without a cane outdoors, and the risk of falling seemed to be low based on the obtained results. However, the presence or absence of falls could not be investigated. In a future study, we will obtain information concerning the history of falls within a 1-year period.

・1st trial data on your FRT may have yielded interesting data. If you have saved this, please compare it and consider it for a future paper.
→We will consider this issue in a future research, as per the reviewer’s suggestion.

・Perhaps in future you could consider movement analysis for FTSST and compare with the movement analysis as described by Sclenkman et al. (future research ideas). You may wish to more clearly outline some of the directions you may want to follow in future research in this topic.

→We would like to thank the reviewer for the suggestion. We will consider this as an issue for future research. Please note that we have discussed this issue as a limitation in the revised manuscript as follows:

“In the FTSST, we assumed that the duration was short and that a momentum transfer strategy would have been used frequently, but it might have been necessary to analyze the movements of the FTSST and investigate the relation of movement patterns. Future studies should consider these aspects when investigating relationship between fall assessment and movement patterns.” (Lines 511–515)

・Your recommendation about using a wall behind the subject performing a FRT is strong and valuable to clinicians in the field. Such recommendations are valuable and perhaps could be made a little more visible.

→Following the reviewer’s suggestion, we have added a part to the Discussion section. The added part is as follows:

“A modified FRT using a wall on the back that limits the use of hip strategies would be the recommended method of evaluation.” (Lines 499–501)

Figures and tables are clear and enhance the paper.
I look forward to seeing this in print.

→We would like to thank the reviewer for the positive comment. We hope that our revisions would meet the reviewer’s expectations and that the revised manuscript is now suitable for publication in your journal.
